# Early antibiotics and risk for necrotizing enterocolitis in premature infants: A narrative review

**DOI:** 10.3389/fped.2023.1112812

**Published:** 2023-02-14

**Authors:** Alain Cuna, Michael J. Morowitz, Venkatesh Sampath

**Affiliations:** ^1^ Division of Neonatology, Children's Mercy Kansas City, Kansas City, MO United States; ^2^ School of Medicine, University of Missouri-Kansas City, Kansas City, MO United States; ^3^ Division of Pediatric General and Thoracic Surgery, Children's Hospital of Pittsburgh of UPMC, Pittsburgh, PA United States; ^4^ School of Medicine, University of Pittsburgh, Pittsburgh, PA, United States

**Keywords:** antibiotic stewardship, intestinal microbiome, prematurity, necrotizing entercolitis, antibiotics, postnatal intestinal adaptation, gut dysbiosis

## Abstract

While prompt initiation of antibiotics at birth due to concerns for early onset sepsis is common, it often leads to many preterm infants being exposed to treatment despite negative blood cultures. Such exposure to early antibiotics can impact the developing gut microbiome putting infants at increased risk of several diseases. Necrotizing enterocolitis (NEC), a devastating inflammatory bowel disease that affects preterm infants, is among the most widely studied neonatal disease that has been linked to early antibiotics. While some studies have demonstrated an increased risk of NEC, other studies have demonstrated seemingly contrary findings of decreased NEC with early antibiotics. Studies using animal models have also yielded differing findings of benefit vs. harm of early antibiotic exposure on subsequent NEC susceptibility. We thus sought to conduct this narrative review to help clarify the relationship between early antibiotics exposure and future risk of NEC in preterm infants. Our objectives are to: (1) summarize findings from human and animal studies that investigated the relationship between early antibiotics and NEC, (2) highlight important limitations of these studies, (3) explore potential mechanisms that can explain why early antibiotics may increase or decrease NEC risk, and (4) identify future directions for research.

## Introduction

Necrotizing enterocolitis (NEC) is a devastating disease that develops in 5%–10% of preterm infants born less than 1500 grams (1). Exaggerated bacteria-induced gut inflammation and necrosis that in severe cases can cause a systemic inflammatory response are considered the central pathogenic mechanism of NEC (2). While the exact mechanisms underlying this exaggerated inflammation remains incompletely understood, prematurity, gut dysbiosis, genetic predisposition, formula-feeding, red blood cell transfusion, and intrauterine growth restriction are considered risk factors (3–5). Because NEC can develop suddenly, addressing risk factors that are potentially modifiable is a key strategy to prevent NEC and help improve outcomes (6). Antibiotics use in the first two weeks of life has been identified as one such risk factor that can potentially modulate risk for NEC (7). Several retrospective studies have demonstrated that early antibiotic use is associated with an increased risk for developing NEC (8–15). Each additional day of antibiotic exposure during the first 7–14 days of life despite sterile blood cultures is estimated to increase the risk for NEC by 7%–20% (8, 9). However, some studies have shown opposite results – that of a protective effect of early antibiotics and NEC. In fact, randomized controlled trials (RCTs) from the late 1970s to late 1990s indicate that prophylactic treatment with oral antibiotics can reduce NEC by half (16–20); and other retrospective studies have demonstrated that early antibiotics is associated with a decrease in NEC incidence compared to infants not exposed to early antibiotics (21–23).

Because of the seemingly contradictory findings from different studies, we sought to conduct this narrative review to help clarify the relationship between early antibiotics and NEC in preterm infants. Our objectives are (1) to summarize human and animal studies investigating early antibiotics and NEC, (2) to highlight challenges and limitations of these studies, (3) to explore mechanisms that may explain how early antibiotics can modify the risk for NEC, and (4) to identify future directions for research.

## Human studies of early antibiotics and NEC

### Randomized studies: old studies indicating that prophylactic early antibiotics may reduce NEC

Five RCTs (16–20) done in the 1970 s-1990 s were conducted to determine whether prophylactic early antibiotics are effective at preventing NEC in preterm infants (Table 1). Oral antibiotics with poor systemic absorption – such as kanamycin, gentamicin, and vancomycin – were used by the studies to limit antibiotic effects to the gastrointestinal tract (24), and were generally administered for 7 to 24 days as enteral feeds were advanced. Overall, a beneficial reduction in NEC with prophylactic early antibiotics was found in four of the five RCTs; and a Cochrane meta-analysis summarizing the 5 trials demonstrated that early antibiotics was beneficial in decreasing NEC by half (RR 0.47, 95% CI 0.28–0.78) (25). Interestingly, beneficial reduction in NEC was observed with antibiotics that targeted gram-negative bacteria (i.e., kanamycin and gentamicin) or gram-positive bacteria (i.e., vancomycin).

**Table 1:** Randomized controlled trials of prophylactic oral antibiotics to reduce NEC.

First Author and Year	Sample size	Intervention	Results
Egan 1976	75	Oral kanamycin vs placebo	Kanamycin decreased NEC (0/35) vs controls (4/40), *p* = 0.038.
Boyle 1978	99	Oral kanamycin vs placebo	NEC rates not different between kanamycin-treated (3/49) and placebo (9/50), *p* = 0.2.
Grylack 1978	42	Oral gentamicin vs placebo	Prophylactic oral gentamicin decreased NEC (0/20) vs placebo (4/22), *p* < 0.05.
Fast 1994	200	Oral gentamicin vs oral IgA-IgG	Oral gentamicin decreased NEC vs oral IgA-IgG (1/100 vs 13/10), *p* = 0.0004.
Siu 1998	140	Oral vancomycin vs placebo	Oral vancomycin decreased NEC (9/71) vs placebo (19/69), *p* = 0.035.

IgA, immunoglobulin A; IgG, immunoglobulin G; NEC, necrotizing enterocolitis.

Despite these positive results, several limitations have dampened adoption of prophylactic early antibiotics to reduce NEC in clinical practice. One limitation is antibiotic resistance. This limitation was demonstrated in the study by Boyle et al. (17) where infants prophylactically treated with kanamycin had higher incidence of kanamycin-resistant enteric gram-negative bacteria compared to controls. A second limitation is selective growth of other pathogenic bacteria (26). This limitation was demonstrated in the study by Siu et. al (20) where infants treated with vancomycin prophylaxis exhibited heavy predominant growth of enteric yeast and gram-negative organisms compared to controls. A third limitation is the questionable generalizability to current clinical practice. These RCTs were done in an era before effective strategies to reduce NEC such as early feeding (27), standardized feeding protocols (28, 29), widespread use of human milk (30, 31) and enhanced infection control practices (32, 33) were part of routine clinical practice. It is thus unknown whether early antibiotics as tested in these early trials would remain effective at reducing NEC in the current setting.

### Retrospective studies: studies that suggest an association between prolonged early antibiotics and NEC

Several retrospective studies have identified a harmful association between early antibiotics and NEC (Table 2). Among the first to report of this harmful association was Cotten et al. (8). Using the Neonatal Research Network (NRN) database, Cotten et al. (8) evaluated 4,039 extremely low birth weight (ELBW) infants who received early antibiotics within 72 h after birth and had sterile blood cultures. The authors found that prolonged early antibiotics for ≥5 days was associated with an increased risk for NEC or death compared to antibiotic treatment for < 5 days (aOR 1.30, 95% CI 1.10–1.54). In another study, Esmaeilizand et al. (12) used data from the Canadian Neonatal Network (CNN) to conduct a matched case-control study of infants with and without NEC. Among the factors they found to be associated with an increased risk for NEC was prolonged early antibiotics (aOR 2.02, 95% CI 1.55–3.13). A population-based study from the Norwegian Neonatal Network also found similar results of higher NEC (aOR 2.27, 95% CI 1.02–5.06) among preterm infants <32 weeks' gestation who were exposed to antibiotics for 3–5 days compared to 0–4 days exposure (34). Other smaller retrospective studies demonstrated how each additional day of empiric antibiotic exposure in the first 7 to 14 days of life can increase the risk for NEC (9, 11, 15) or the composite outcome of NEC + late-onset sepsis + death (10, 13). Taken together, these studies seem to suggest that prolonged treatment with early antibiotics despite negative blood cultures can increase the risk for NEC and other poor outcomes (Table 2).

**Table 2:** Retrospective studies showing the association between prolonged early antibiotics and NEC.

First Author and Year	Study design	Study Population	Results
Cotten 2009	Multi-center retrospective cohort study	4,039 ELBW infants treated with early antibiotics despite sterile cultures. Infants who received ≥5 days early antibiotics were compared to infants who received <5 days.	Increased odds for death (1.46, 95% CI 1.19-1.78) and increased odds for NEC or death (1.30, 95% CI 1.10-1.54) associated with ≥5 days exposure to early antibiotics.
Alexander 2011	Single-center retrospective case-control study	124 NEC cases (stage 2 or 3) were compared to 248 controls that were matched by gestational age, birth weight, and birth year.	Cumulative duration of antibiotic exposure associated with increased risk of NEC (aOR 1.10, 95% CI 1.02-1.19).
Kuppala 2011	Multi-center retrospective cohort study	365 VLBW infants ≤ 32 weeks’ gestation exposed to early antibiotics despite sterile cultures. Infants were categorized into prolonged antibiotics (≥5 days), limited antibiotics (1–4 days) and no antibiotics (0 days).	Each day of early antibiotic treatment was associated with increased odds for composite outcome of NEC, LOS, and death (aOR 1.24, 95% CI 1.07-1.44).
Ghany 2012	Single-center retrospective cohort study	207 VLBW infants who received early antibiotics despite sterile cultures. Antibiotic treatment for ≥5 days were compared to <5 days.	Each day of early antibiotic treatment was associated with increased odds of NEC (aOR 1.32, 95% CI 1.05-1.65).
Cantey 2018	Single-center retrospective cohort study	374 VLBW infants with gestational age <33 weeks at birth. Infants with composite outcome of interest (NEC + LOS + death) were compared to infants without this composite outcome.	Each day of early antibiotic treatment in the first 14 days of life was associated with increased risk for the composite outcome of NEC + LOS + death (aOR 1.24, 95% CI 1.17-1.31).
Esmaeilizand 2018	Multi-center retrospective case-control study	224 NEC cases (stage 2 or 3) were compared with 447 controls that were matched by gestational age, birth weight, and gender.	Early antibiotic treatment for ≥5 days was associated with increased NEC (aOR 2.02, 95% CI 1.55-3.13) compared to antibiotic treatment for 0–4 days.
Raba 2019	Single-center retrospective case-control study	22 NEC cases (stage 2 or 3) were compared with 32 controls that were matched by gestational age, sex, maternal chorioamnionitis exposure, and mode of delivery.	Prolonged early antibiotics for >5 days associated with NEC (OR 3.6, 95% CI 1.13-11.47).
Chen 2022	Single-center retrospective cohort study	132 VLBW infants were investigated by multivariable logistic regression to determine the association of antibiotic treatment and NEC.	Each day of early antibiotic treatment in the first 14 days of life was associated with increased odds of NEC (aOR 1.28, 95% CI 1.03-1.59).
Zhu 2022	Single-center retrospective cohort study	51 NEC cases (stage 2 or 3) were compared with 516 with no NEC. Infants were all VLBW and <32 weeks’ gestation at birth.	Early antibiotic therapy duration was associated with increased odds of NEC (aOR 1.27, 95% CI 1.13-1.42).
Vatne 2022	Population-based retrospective study	4,932 VLBW infants were studied using nationwide registry of Norway. Association between empirical antibiotics and NEC was assessed using multivariable logistic regression models, adjusting for known confounders.	Antibiotics ≥ 5 days were associated with higher odds of NEC (aOR 2.27, 95% CI 1.02-5.06).

ELBW, extremely low birth weight; NEC, necrotizing enterocolitis; LOS, late-onset sepsis; VLBW, very low birth weight.

A major limitation of these retrospective studies is confounding by indication that comes from the possibility that prolonged early antibiotics is simply a marker of illness severity. In the majority of the studies, infants treated with prolonged early antibiotics were also more premature, had lower birth weight, and more likely to be born in the setting of chorioamnionitis compared to infants treated for <5 days (8–10, 13). It is well-known that the incidence and severity of NEC is inversely correlated to prematurity and birth weight (35, 36). Moreover, maternal chorioamnionitis is an important risk factor for early-onset sepsis that often informs the decision to use early antibiotics treatment and has also been shown to increase risk for NEC (37). It is thus unclear whether it is prolonged early antibiotics or these differences in underlying baseline characteristics that truly increases risk for NEC. Efforts to control for these differences, such as by propensity matching or logistic regression, are likely not able to fully adjust for the impact of these differences in NEC risk.

### Retrospective studies: studies that suggest a potential protective effect of limited early antibiotics against NEC

Other retrospective studies have demonstrated contrary findings of a protective association between early antibiotics and NEC (Table 3). The first two studies to report of this protective association were small, case control studies with approximately 200 to 350 infants (38, 39). Krediet et al. (38) conducted a matched case-control study (*n* = 208 infants) to identify risk factors that may explain an increase in NEC incidence at their local institution. The authors found that treatment with antibiotics within 48 h after birth was associated with a reduction in NEC (OR 0.3, 95% CI 0.2–0.6). Berkhout et al. (39) also conducted a matched case-control study (*n* = 336 infants) and found a similar association of decreased NEC with early antibiotics. Three subsequent studies (21–23) were large, multi-center studies with approximately 1,200 to 14,000 infants. The largest of these studies was Ting et al. (22) (*n* = 14,207 infants). Using data from the CNN, Ting et al. (22) investigated the impact of early antibiotics on neonatal outcomes and found that limited early antibiotics (≤3 days) was associated with a reduction in NEC compared to untreated controls (aOR 0.74, 95% CI 0.55–0.99). The second largest of these studies was Li et al. (21) (*n* = 2,831 infants). Using prospective data collected from 13 neonatal intensive care units from five continents, Li et al. found that NEC incidence was lower among infants treated with early antibiotics compared to infants with no antibiotic exposure (aOR 0.25, 95% CI 0.12–0.47). Lastly, Dierikx et al. (23) studied 1,259 very low birth weight (VLBW) infants from 9 centers in the Netherlands and Belgium and found that early antibiotics was associated with decreased risk for NEC compared to no antibiotics (aOR 0.47, 95% CI 0.23–0.96).

**Table 3:** Retrospective studies suggesting that limited early antibiotics decreases risk for NEC.

First Author and Year	Study design	Study Population	Results
Krediet 2003	Single-center matched case-control study	104 NEC cases (stage 2 or 3) were compared to 104 controls matched by gestational age, birth weight, and period of admission.	Antibiotic treatment <48 h after birth was associated with decreased risk for NEC (aOR 0.3, 95% CI 0.2-0.6).
Berkhout 2018	Multi-center matched case-control study	56 NEC cases (stage 2 or 3) were compared to 280 controls that were matched by gestational age, birth weight, and postnatal age of NEC. Infants with 1–3 days and >3 days of antibiotics were compared to infants with no antibiotics as reference.	Decreased NEC occurrence was associated with antibiotic exposure for 1–3 days (aOR 0.21, 95% CI 0.08-0.54) and >3 days (aOR 0.23, 95% CI 0.08-0.65).
Ting 2019	Multi-center retrospective cohort study	14,207 VLBW infants with sterile cultures were divided based on antibiotic exposure of 0 days, 1–3 days, and 4–7 days.	Infants exposed to limited antibiotics for 1–3 days have lower odds of NEC (aOR 0.74, 0.55–0.99) than infants who did not receive any antibiotics.
Li 2020	Multi-center retrospective cohort study	2,562 VLBW infants who received early antibiotics within 72 h after birth were compared to 269 VLBW infants who did not receive early antibiotics.	NEC incidence was lower in infants who received early antibiotics (aOR 0.57, 95% CI 0.35-0.94).
Dierikx 2022	Multi-center retrospective cohort study	1,259 infants <30 weeks’ gestation with sterile cultures were divided into no antibiotics, short antibiotics exposure (≤3 days), and long antibiotics exposure (>3 days).	Short antibiotic exposure had decreased NEC incidence compared to long antibiotic exposure (aOR 0.58, 95% CI 0.35-0.96) and no antibiotic exposure (aOR 0.39, 95% CI 0.19-0.80).

NEC, necrotizing enterocolitis; VLBW, very low birth weight infants.

Analysis based on duration of treatment provided additional insights regarding the relationship between early antibiotics and NEC. In the CNN study (22), Ting et al. divided the study cohort based on duration of antibiotic treatment (0 days vs. ≤3 days vs. >3 days). The authors found that limited early antibiotics (≤3 days) was associated with a reduction in NEC compared to untreated controls (0.74, 95% CI 0.55–0.99); but prolonged early antibiotics (>3 days) was not associated with either increased or decreased NEC risk when compared to either 0 days or ≤3 days. Dierikx et al. (23) also performed additional analysis based on duration of treatment and found similar results of protective effects of limited early antibiotics given for ≤3 days; whereas prolonged early antibiotics (>3 days) was neither harmful nor protective. While these two studies suggest that a limited course of early antibiotics (≤3 days) may help reduce the risk for NEC in preterm infants, the study by Vatne et al. (34) had different results. In their large population-based study, limited treatment with early antibiotics for 1–3 days did *not* have a protective effect compared to untreated controls (aOR 2.02, 95% CI 0.22–18.3).

A potential limitation of studies suggesting that limited early antibiotics can protect against NEC is the use of infants with no antibiotic exposure as the reference group. This limitation was suggested by Berkhout et al. (39) as another form of confounding by indication that arises from the possibility that infants with no antibiotic exposure represent an underrecognized population with high baseline risk for NEC. In the three large multi-center studies referenced above, infants with no antibiotic exposure were more likely to be small for gestational age (SGA) and born by caesarian section without premature rupture of membranes compared to infants treated with early antibiotics (21–23). These differences in baseline characteristics suggest that infants with no antibiotic exposure were born prematurely due to poor fetal Dopplers and intrauterine growth restriction which, while considered low-risk for early-onset sepsis (40, 41), are associated with higher risk for NEC (42, 43). Thus, there is a possibility that using infants with “no antibiotic exposure” as the reference may make it appear that early antibiotics is protective against NEC.

## Animal studies investigating the relationship of early antibiotics and NEC

Given the varying results and important limitations of existing studies in humans, studies using animal models have been conducted to provide mechanistic insights on the effects of early antibiotics on the developing neonatal gut. In this section, we will review findings from two experimental animal models of early antibiotics and NEC, explore potential mechanisms that explain their results, and discuss the differences and limitations of each model.

### Piglet model of early antibiotics and NEC

The first animal model used to investigate the effects of early antibiotics on the newborn gut was the preterm pig model of experimental NEC. In this model, pigs that were delivered prematurely *via* caesarian section at ∼92% gestation and transitioned gradually from parenteral to enteral nutrition over the next 5 days develop experimental NEC spontaneously (44). But when early antibiotics were administered concurrently starting from birth until time of sacrifice, substantial protection from NEC among antibiotic-treated pigs was demonstrated compared to untreated controls (45, 46). Interestingly, the protective effects against NEC were limited to when antibiotics were given orally and not parenterally (47, 48) – a finding that mirrors early RCTs of prophylactic early antibiotics (25). Thus, studies using this piglet model provide evidence supporting findings from human studies which suggest that early antibiotics is protective against NEC.

However, it is important to note that more recent investigation (49) with the piglet model have identified important adverse effects of early antibiotics, including emergence of antibiotic-resistant gut organisms and suppression of systemic immune function. Combining oral antibiotics with fecal microbiota transplantation did not prevent the adverse effects of oral antibiotics as hypothesized, suggesting pervasive effects of antibiotics on immune function. Thus, while early antibiotics were protective against NEC in piglets, important adverse effects were also found that warrant further investigation.

### Mouse model of early antibiotics and NEC

Another animal model used to investigate early antibiotics and NEC was the newborn mouse model of NEC. In this model, newborn mice delivered naturally at term were immediately exposed to 10 days of systemic antibiotics (50). After a washout period of 4 days, the pups were then exposed to oral bacterial challenge with *Klebsiella spp*. to induce NEC. The authors found that NEC-like intestinal injury was significantly worse in antibiotic-treated pups compared to untreated controls. Thus, in contrast to the piglet model, studies using the newborn mouse model provide evidence supporting findings from other human studies which suggest that early, prolonged, systemic exposure to antibiotics increases risk for NEC.

### Differences between piglet and mouse model of early antibiotics and NEC

Several experimental differences between the piglet and mouse models may explain the opposing findings from animal studies (Supplementary Table). One difference is with regards to the duration of early antibiotics. In the piglet model, piglets were treated for only 5 days of antibiotics, whereas in the mouse model, pups were treated for 10 days. Modulating effects of antibiotic duration on intestinal injury can be seen in experiments with adult mice, where 4 days resulted in transient ileal injury that quickly reverses by stopping treatment (51), whereas 14 days of antibiotics caused several more intestinal impairments including gut dysbiosis, reduced short-chain fatty acid concentrations, disrupted intestinal tight junction barrier, and increased activation of autophagy (52). Additional experimental studies that vary duration of early antibiotics within the same animal model may help better elucidate the impact of duration of treatment on NEC susceptibility. Another difference that can explain these opposing findings is the differences in gestational age used in each animal model. In the piglet model, piglets were born *via* caesarian section at 92% gestation, whereas the mouse model used mouse pups born naturally at term gestation. Thus, it is possible that the experiments in pigs modeled the effects of early antibiotics in preterm infants, while the mice experiments modeled the effects of early antibiotics in term infants. Other differences that could explain the opposing findings between the two animal models include differences in route of antibiotic administration, method of induction of experimental NEC, and the presence or absence of a wash-off period from antibiotics before NEC induction (Supplementary Table).

## Proposed mechanisms by which by early antibiotics might increase or decrease the risk of NEC

### Delayed bacterial colonization allows preterm gut defenses to mature and decreases NEC risk

**Figure 1:**
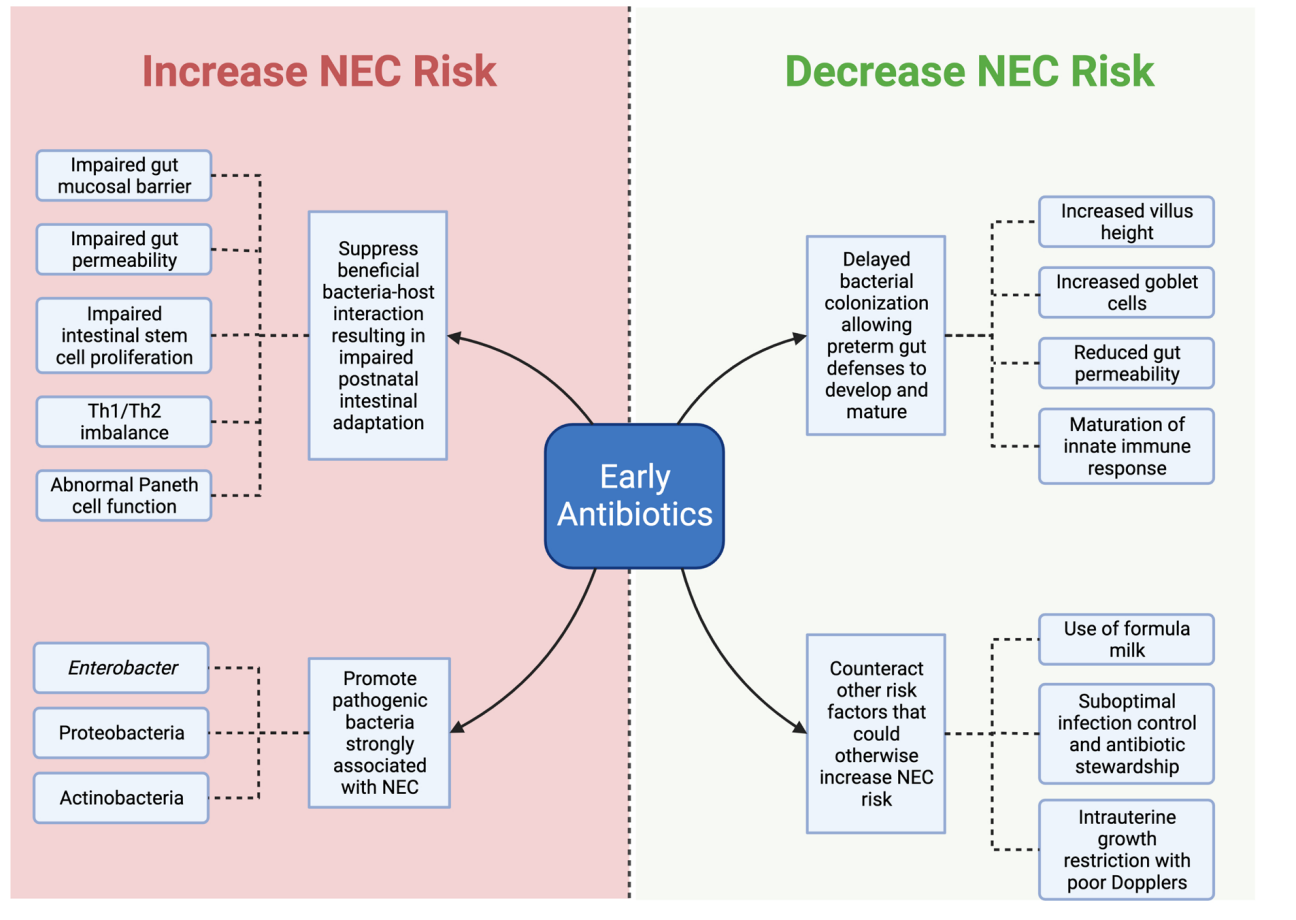
Diagram of potential mechanisms by which early antibiotics can increase or decrease NEC risk. Created with Biorender.com.

Delayed bacterial colonization is hypothesized as the main mechanism by which early antibiotics protect against NEC (Figure 1). Studies in mice as well as human samples from immature intestine have shown that the preterm gut is inherently predisposed towards excessive inflammation (53–55). Delaying gut colonization can potentially allow more time for the preterm gut defenses to develop and mature before encountering bacteria, viruses, and fungi that can otherwise trigger pathologic intestinal inflammation and NEC. In a physiologic study of infants, intestinal permeability was higher in preterm compared to term infants only in the first 2 days of life. By 3 to 6 days of life, intestinal permeability between preterm and term infants was already similar, suggesting rapid postnatal adaptation of preterm intestinal mucosal barrier (56). A limited course of early antibiotics during the first few days after preterm birth may thus be sufficient to achieve this delayed colonization and allow maturation of preterm gut defenses without harming the developing gut microbiome (57). This hypothesis is further supported by the several improvements in intestinal structure, function, and immunity that have been identified in preterm pigs treated with early antibiotics for the first 5 days of life. These include increased villus height, higher digestive enzyme activity, increased goblet cell density, reduced gut permeability, downregulation of genes related to inflammation and innate immune response, and upregulation of genes related to metabolism (45–48). Thus, there is supporting evidence – from experimental piglet studies and from older RCTs of preterm infants – that early antibiotics can be protective against NEC by delaying bacterial colonization and allowing the immature intestine to better adapt to postnatal milieu.

### Aberrant gut colonization disrupts proper postnatal intestinal adaptation and increases NEC risk

On the other hand, aberrant gut colonization is the main mechanism by which early antibiotics is hypothesized to increase risk for NEC (Figure 1) (58). Early antibiotics can predispose to aberrant gut colonization in a few ways. One is by suppression of beneficial bacteria that contribute to the physiologic development of the postnatal gut (59, 60). While beyond the scope of this review, several studies have demonstrated that synergistic relationships between colonizing microbes and the host gut mucosa are crucial for successful postnatal intestinal adaptation (61–63). For example, studies in mice reveal that the interaction of commensal bacteria with intestinal TLR signaling plays a critical role for maintaining intestinal epithelial homeostasis and helps protect against gut injury (61). In another study, gut colonization of mice with the symbiotic bacteria *Bacteroides fragilis* was found to mediate establishment of proper Th1/Th2 balance through bacterial surface polysaccharide A (62). Disruptions to this normal process of gut colonization with commensals – such as with early antibiotic use – can thus lead to a dysfunctional gut mucosa predisposed to NEC (64–66). This hypothesis is supported by the mouse model by Chaaban et al. (50) where exposure of newborn pups to 10 days of antibiotics resulted in several impairments to gut mucosal barrier, intestinal permeability, intestinal stem cell proliferation, and Paneth cell function.

Another way by which early antibiotics cause aberrant gut colonization is by increasing the population of potentially pathogenic bacteria. Next-generation sequencing of stools from preterm infants demonstrated how antibiotic treatment is associated with increased relative abundance of *Enterobacter*, Proteobacteria, Actinobacteria in conjunction with decreased relative abundance of Firmicutes and Bacteroidetes (67–69). Moreover, this abnormal pattern of increased Proteobacteria and decreased Firmicutes and Bacteroidetes have been identified in gut microbiota studies to precede development of NEC in preterm infants (70–73). In animals, landmark studies have shown how antibiotic-treated animals but not untreated controls are susceptible to pathogenic bacterial challenges (74, 75), partly due to loss of colonization resistance afforded by commensals (76).

Thus, there is also supporting evidence – originating from mouse models as well as infant gut microbiome studies – that early antibiotics can be harmful to the developing neonatal gut by increasing pathogenic bacteria at the expense of beneficial commensals.

## Additional speculations from human and animal studies

### Is there an interaction between early antibiotics and other risk factors of NEC?

NEC is multi-factorial in origin, and early antibiotics exposure is only one of several risk factors that could modify NEC risk. One speculation is that perhaps harm or protection against NEC can depend on the interaction of early antibiotics with other risk factors of NEC (77, 78). For example, feeding with formula is a strong risk factor for NEC that is known to alter the developing gut microbiome; whereas feeding with human milk is protective and promotes colonization with beneficial commensals (79). It is thus possible that early antibiotics is protective when formula feeding is prevalent such as during RCTs of the 1970 s-1990 s; but is now harmful in the current era when human milk is the feeding standard for preterm infants. In addition to the type of milk, variation in advancement of feeding in preterm infants could also play a confounding role in determining the impact of early antibiotics and NEC (80).

Another risk factor for NEC is intestinal colonization with harmful pathogens from the NICU environment. In the study by Li et al. (21), about half of the cohort came from Asia where antibiotic stewardship and infection control practices can be a challenge (81–83), and nosocomial infection with resistant strains is high (84–86). It is thus possible that early antibiotics is protective in NICU environments where the local antibiogram has a predominance of pathogenic and resistant bacterial isolates. However, the protective association of early antibiotics and NEC was also found in studies from developed counties such as Canada and Europe, where antibiotic stewardship and infection control practices are more robust. This consistency across developed and developing countries suggest that the beneficial effect of early antibiotics remain despite differences in these factors.

Another important factor that can interact with early antibiotics is supplementation with probiotics. While beyond the scope of this review, there is extensive literature supporting the protective effects of probiotics against NEC in general (87, 88). Looking specifically at the interaction of early antibiotics and probiotics, one study showed that antibiotic-treated mice supplemented with probiotics exhibited a reduction in pathogenic *Enterobacteriaceae* while promoting growth of commensal *Firmicutes* compared to antibiotic-treated mice with no probiotic supplementation (89). Similarly, in a prospective observational study, extremely preterm infants with high antibiotic exposure that also received probiotics had comparable microbial diversity and antibiotic resistome as more mature infants, suggesting that probiotic supplementation may have alleviated the harmful effects of antibiotics on the gut microbiota (90). Other factors that might interact with early antibiotics to modify future NEC risk include prior maternal exposure to antibiotics (91–93), genetic predisposition to NEC (4), and genetic predilection for antibiotic resistance (94).

### Is limiting activity of early antibiotics key?

We also speculate that perhaps limiting antibiotic activity may be the key for reaping benefits of early antibiotics on NEC risk without harm. The early clinical trials that showed benefit of prophylactic antibiotics used oral agents with narrow spectrum and poor systemic absorption that limited antibiotic activity to the gastrointestinal tract (25). On the other hand, more recent studies that used broad-spectrum antibiotics given intravenously as part of clinical care seem to suggest that a limited exposure of less than 3 to 5 days can decrease subsequent risk for NEC (22, 23, 39). In animal models, prolonged treatment for 10 days with antibiotics resulted in several intestinal impairments and increased NEC severity compared to controls (50) but limited treatment for 5 days with poorly absorbed oral antibiotics caused improved maturation of preterm gut defenses and decreased NEC (45).

Studies that investigated the effects of antibiotics on gut microbiome also provide evidence that limited early antibiotics may not be as harmful as previously thought. In one study, Zwittink et al. (95) obtained fecal samples from preterm infants with no, short (≤3 days), or long (≥5 days) treatment with antibiotics. 16S rRNA sequencing revealed that while both short and long antibiotic treatment significantly lowered the abundance of the commensal *Bifidobacterium*, quick recovery of *Bifidobacterium* abundance was observed among infants exposed to short antibiotics while infants exposed to long antibiotics exhibited a persistent reduction of *Bifidobacterium*. In another study, Kim et al. (57) randomized preterm infants at low risk for sepsis to receive 2 days of placebo vs. ampicillin and gentamicin, analyzed their fecal microbiome, and administered early fecal supernatant to pregnant gnotobiotic mice. Surprisingly, in this study limited treatment with 2 days of antibiotics did not alter the fecal microbiome of treated infants compared to placebo; and pups of gnotobiotic pregnant mice exposed to the fecal supernatant of antibiotic-treated infants did not have any differences in gut microbiome, weight gain, and markers of intestinal health compared to controls.

Thus, there is evidence from both human and animal studies to suggest that limiting early antibiotics – whether by using narrow-spectrum, poorly absorbed oral antibiotics that limit activity in the intestinal tract, or by using broad-spectrum intravenous antibiotics but treating for shorter periods of time – may not be harmful and may have some benefit in decreasing NEC risk. One important caveat about poorly absorbed oral antibiotics in preterm infants is that in some studies, substantial systemic concentrations of these oral antibiotics can be found in the serum, especially when given in the first few days of life (96).

### Do antibiotics have direct effects on host immunity and inflammation?

It is also possible that antibiotics have direct effects on immune cells and immune-mediated receptors that can modify risk for NEC (97, 98). For example, *in vitro* studies revealed that gentamicin, a first-line antibiotic drug of choice for neonatal sepsis, can directly inhibit the chemotactic response of human polymorphonuclear leukocytes (99). In another study, mice given Ampicillin or Vancomycin, two other antibiotics commonly used in neonates, exhibited significant downregulation of Th17-related genes in the ileum (100). In the piglet model of NEC, 5 days of antibiotic treatment resulted in significant downregulation of genes related to inflammation and innate immune response following compared to controls (45). Recent studies also suggest that antibiotic-induced elimination of bacterial pathogens can elicit the release of microbial components such as LPS that further worsens inflammation (101, 102). While it is difficult to discern whether these immune changes are independent of antibiotic-induced alterations in gut microbiome, there is accumulating evidence that antibiotics can have direct effects on host immunity and inflammation which may impact disease (103).

## Summary and future directions

Although human and animal studies seem to suggest that treatment with early antibiotics can alter future risk for NEC (Table 4), inherent limitations of these studies must also be carefully considered for proper interpretation. RCTs done several decades ago with oral, non-absorbable, and narrow-spectrum antibiotics showed a reduction in NEC, but the relevance of such studies to modern NICU practice is uncertain. A more recent RCT of prophylactic intravenous antibiotics for 5 days vs. no antibiotics did not find any benefit with prophylactic antibiotics, but the study included low-risk infants (median gestational age 34 weeks) and was not powered to detect differences in NEC (*N* = 140) (104). Retrospective cohort studies suggest that prolonged duration of early antibiotics (>3 to 5 days) can increase risk for NEC, but these studies present only low quality of evidence as there is significant confounding by indication of antibiotic use and unequal exposure to other NEC-associated risk factors. Other retrospective studies suggest that limited duration of antibiotic use (<3 to 5 days) may decrease NEC risk, but these studies should also be interpreted with caution as using infants with no antibiotic exposure as reference may be a source of confounding bias. Interestingly, some animal studies seem to mimic human data with regards to duration of antibiotics and NEC risk, but additional experimentation to evaluate the impact of several other important variables – such as gestational age and mode of NEC induction – is needed. Of note, none of the human studies and few of the animal studies examined the effects of early antibiotics on the gut microbiome, further limiting mechanistic interpretation of results.

**Table 4:** Summary of human and animal studies regarding early antibiotics and NEC.

Study Design	Increased risk of NEC	Decreased risk of NEC	No difference in NEC
Randomized clinical trials		Egan 1976 Grylack 1978 Fast 1994 Siu 1998	Boyle 1978 Tagare 2010
Retrospective clinical studies	Cotten 2009 Alexander 2011 Kuppala 2011 Ghany 2012 Cantey 2018 Esmaeilizand 2018 Raba 2019 Chen 2022 Zhu 2022 Vatne 2022	Krediet 2003 Berkhout 2018 Ting 2019 Li 2020 Dierikx 2022	Greenberg 2019
Animal studies	Chaaban 2022	Sangild 2006 Jiang 2012 Jensen 2014 Nguyen 2016 Birck 2016	

Additional studies in humans and animals are needed to attain a better understanding of the effects of early antibiotics on later NEC risk. Studies that evaluate effects of early antibiotics in intestinal immunity should also evaluate parallel changes in the gut microbiome. As stool samples may only reflect changes in either colonic mucosa or transient luminal contents, animal studies should endeavor to obtain intestinal mucosal samples from different parts of the intestinal tract to accurately investigate host and microbiota changes induced by antibiotics in the gut mucosa. In addition to antibiotic-induced changes on the gut microbiome, additional research into the direct effects of antibiotics on intestinal immunity is also needed. Ultimately, the inherent limitations of existing human studies warrant large prospective RCTs (105) as well as well-designed prospective observational studies (106) to study the impact of withholding early antibiotic use or limiting duration of exposure on NEC as well as other outcomes including late-onset sepsis and bronchopulmonary dysplasia. The NICU Antibiotics and Outcomes Trial (NANO) as well as other studies are beginning to address this question (106–108). Future clinical practice on early antibiotics use will likely be impacted by these ongoing studies. In the meantime, current efforts to implement sound antibiotic stewardship practices in the NICU should be followed (109, 110). This includes limiting prophylactic administration of early antibiotics only to infants with strong concerns for early-onset sepsis, such as those with prolonged rupture of membranes or maternal chorioamnionitis (40, 41). Antibiotics should also be promptly discontinued once blood cultures remain sterile for 24 to 48 h (111). Prolonged use of early antibiotics in the absence of positive blood cultures should be discouraged.
