# Supplementary material for: Early antibiotics and risk for necrotizing enterocolitis in premature infants: A narrative review
Source: Front Pediatr. 2023 Feb 14;11:1112812. doi: 10.3389/fped.2023.1112812 (PMC9971631; doi:10.3389/fped.2023.1112812)
Supplement: Supplementary file 1 [file Table1.docx]

**Supplemental Table**. Major differences between the two animal models investigating early antibiotics and NEC.

|  | **Piglet model** | **Mouse model** |
| --- | --- | --- |
| **Gestational age** | Preterm | Term |
| **Antibiotic duration** | 5 days | 10 days |
| **Route of administration** | Oral | Intraperitoneal |
| **NEC induction** | Formula-feeding | Oral bacterial challenge |
| **Wash-off period before NEC induction** | No | Yes |
